# The Effect of Large Doses of Cocaine upon the Central Nervous System

**Published:** 1887-03

**Authors:** 


THE EFFECT OF LARGE DOSES OF COCAINE UPON THE CENTRAL NERVOUS SYSTEM.—Dr. Bey, of Cairo, has had the following experience in the use of cocaine as a means of checking the opium-habit. He began with doses of five centigrammes, three or four times daily, but the patient's sensations of relief and stimulation were so pleasurable that the patient soon established a cocaine habit. He sought relief for each slight ailment in an injection of cocaine, the dose increasing until half a gramme, and even $\frac{8}{10}$ of a gramme, was taken secretly daily. This produced loss of appetite, great irritability, ringing in the ears, and, from time to time, dyspnœa and hallucinations of sight and hearing. These unpleasant symptoms the patient had learned to relieve by injections of morphine, until he became skilled in the antagonistic use of the drugs. An attack of herpes and its neuralgic pains drove him to double his doses of cocaine until for two or three days he took a gramme, and at times one and a-half grammes daily. Then followed a condition very like delirium tremens—tremors, lack of muscular tonicity, incontinence of urine, alterations in the nails of the fingers and toes, the greatest agitation, severe hallucinations of sight, hearing, and smell, injected conjunctivæ, a staring expression. He fired a pistol at imaginary objects, attacked his servant, and was finally placed under hospital restraint. Here he soon recovered under morphine injections of five centigrammes, three times daily.

